# Representation of Ecosystem Services by Terrestrial Protected Areas: Chile as a Case Study

**DOI:** 10.1371/journal.pone.0082643

**Published:** 2013-12-20

**Authors:** América P. Durán, Stefano Casalegno, Pablo A. Marquet, Kevin J. Gaston

**Affiliations:** 1 Environment and Sustainability Institute, University of Exeter, Penryn, Cornwall, United Kingdom; 2 Departamento de Ecología, Facultad de Ciencias Biológicas, Pontificia Universidad Católica de Chile, Santiago, Chile; 3 Instituto de Ecología y Biodiversidad (IEB), Santiago, Chile; 4 Santa Fe Institute, Santa Fe, New Mexico, United States of America; 5 Laboratorio Internacional en Cambio Global (LINCGlobal), Facultad de Ciencias Biológicas, Pontificia Universidad Católica de Chile, Santiago, Chile; University of Saskatchewan, Canada

## Abstract

Protected areas are increasingly considered to play a key role in the global maintenance of ecosystem processes and the ecosystem services they provide. It is thus vital to assess the extent to which existing protected area systems represent those services. Here, for the first time, we document the effectiveness of the current Chilean protected area system and its planned extensions in representing both ecosystem services (plant productivity, carbon storage and agricultural production) and biodiversity. Additionally, we evaluate the effectiveness of protected areas based on their respective management objectives. Our results show that existing protected areas in Chile do not contain an unusually high proportion of carbon storage (14.9%), agricultural production (0.2%) or biodiversity (11.8%), and also represent a low level of plant productivity (Normalized Difference Vegetation Index of 0.38). Proposed additional priority sites enhance the representation of ecosystem services and biodiversity, but not sufficiently to attain levels of representation higher than would be expected for their area of coverage. Moreover, when the species groups were assessed separately, amphibians was the only one well represented. Suggested priority sites for biodiversity conservation, without formal protection yet, was the only protected area category that over-represents carbon storage, agricultural production and biodiversity. The low representation of ecosystem services and species’ distribution ranges by the current protected area system is because these protected areas are heavily biased toward southern Chile, and contain large extents of ice and bare rock. The designation and management of proposed priority sites needs to be addressed in order to increase the representation of ecosystem services within the Chilean protected area system.

## Introduction

Ecosystem services, the benefits that humans derive from ecosystems, are vital for sustaining human well-being [1]–[3]. However, these services are also increasingly threatened by human activities [1]. It is thus critical to evaluate to what extent current conservation strategies capture ecosystem services, and therefore might ensure their provision in the future [4], [5]. Due to their vast terrestrial coverage and historical success in conserving natural ecosystems, protected areas are increasingly considered to play a key role in the maintenance of the ecosystem processes that promote ecosystem service provision [1], [6], [7]. However, most existing protected areas have not been designated, established or managed to meet this specific objective, and might reasonably be expected in some instances to be inappropriate for doing so (e.g. agricultural and timber production). Indeed, whilst the representation of biodiversity within protected areas has been widely assessed [8]–[13], only a few studies have evaluated to what extent these are capturing ecosystem services [14]–[16]. Moreover, those studies that have been conducted have tended to focus on the representation of a single ecosystem service [16], or have been carried out at a rather coarse spatial resolution [15]. Assessments considering multiple services at a finer resolution are limited to developed countries (i.e. highly human-dominated regions) [17]. A broader range of studies are required to help understand the nature of the gaps in ecosystem service conservation and where they occur, and thus to aid systematic planning to designate and establish future protected areas to redress these gaps.

The provision of key ecosystem services can present trade-offs (e.g. carbon storage vs agricultural production) making their conservation within the same areas challenging [18], [19]. Ecosystem services that involve active management practices can influence the potential for “disservices”, often harming biodiversity and reducing the production of other services. For example, agriculture is a highly valuable provisioning service [1], providing food, forage, fibre, bioenergy and pharmaceuticals, but due to the often intensive form of associated land management, it is commonly considered a negative pressure on biological conservation [20], [21]. How well protected area systems represent ecosystem services will depend, therefore, on what services are considered valuable to include or exclude, and this threshold is generally determined by the socioeconomic conditions and climatic region in which the protected area system is located. For instance, in a highly human-dominated region, where a higher proportion of land has been converted and species assemblages may have long been shaped by human activities, agriculture might be promoted, or at least tolerated, as an ecosystem service within protected areas [17]. In contrast, in a less developed region with relatively pristine ecosystems, this activity might be excluded from protected areas [22].

There is a particular paucity of data for appropriate evaluation of protected area effectiveness in capturing ecosystem services in poor and developing countries [23]. These are often also countries that are particularly rich in natural resources (renewable and non-renewable) and whose economies depend on their extraction, which makes the establishment of protected areas, strict management objectives and the assessment of their performance particularly challenging. Chile provides one such example [24], [25]. Its economy depends strongly on extractive activities such as wood pulp production, agricultural production and mining, and the establishment and performance of strict environmental management strategies has been poor [24], [26]. Indeed, the Chilean National System of Protected Areas (SNASPE) is known to be inefficient in providing adequate coverage of the country’s biodiversity [27]–[30] and is underfunded, receiving only 0.03% of the national budget [CONAF 2005, unpublished data]. In response, the Chilean Ministry of Environment has made an urgent call to assess and improve the current protected area system [31] and to increase the protection of the country’s non-transformed ecosystems. Thus, in collaboration with the Global Environment Facility (GEF) and the United Nations Development Program (UNDP), the Ministry of Environment aims to create an integrated public and private protected area system in order to increase protected area coverage and share responsibilities and costs among the different governmental and private bodies [31]. Private protected areas and priority sites for biodiversity conservation have been identified and suggested to be incorporated into a new integrated protected area system, however the extent to which these locations are valuable for ecosystem service provision is unknown.

This study analyses for the first time to what extent the current and suggested integrated protected area system represent selected ecosystem services of Chile. Specifically, the representation of three ecosystem services - plant productivity, carbon storage, agricultural production - and biodiversity is assessed under three protection scenarios. These scenarios capture the current status and medium-term projections for the protected area system. Given the large extent of pristine forest ecosystem remaining, we would expect that Chilean protected areas tend to represent high levels of net primary production, carbon storage, and biodiversity, but tend to exclude agricultural production. We address three main questions: 1) How are the three ecosystem services and biodiversity distributed across Chile?; 2) To what extent do the three protection scenarios represent the chosen ecosystem services and biodiversity?; and 3) How effective are Chilean protected area categories in representing ecosystem services and biodiversity?

## Data and Methods

### Protected Area System Coverage

In order to assess the effectiveness of the current Chilean protection system we considered all areas with statutory protection. These are the protected areas belonging to SNASPE, which comprises 33 national parks, 49 national reserves, and 16 natural monuments. We also included nature sanctuaries (n = 31), and lands protected by the Chilean Ministry of National Heritage (n = 18) (Table S1). SNASPE makes up the majority of traditional public protected areas in Chile and is administered by the National Forestry Corporation (CONAF) created by the Chilean government in 1984. Nature sanctuaries include both public and private lands that obtained statutory protection under the Chilean National Environmental Law in 1994 and law No. 17 288 relating to National Monuments in 1970. Those lands administered by the Ministry of National Heritage are public protected areas managed exclusively for conservation and established by decree in 1977. To assess the potential effectiveness of the new suggested sites we considered protected priority sites for biodiversity conservation (PSBC) identified by the National Environmental Commission (CONAMA) in 2011 (n = 68), and private protected areas (n = 295). PSBC identified by the Chilean Ministry of Environment are part of the countrýs National Biodiversity Strategy [CONAMA 2003, unpublished data], which aims to improve the representation of biodiversity within the Chilean protected area system. Private protected areas were also defined by the National Environmental Law (Article 35), and these are portions of private land which the owners have voluntarily set aside for conservation objectives. Both PSBC and private protected areas have not received statutory protection yet, but they are suggested as protected areas to be incorporated into the current protected area system and thus create the new *integrated protected area system*. Current protected areas, PSBC and private protected areas datasets were obtained from the Chilean Ministry of Environment in vector format. Current protected areas datasets are freely available at the Chilean Ministry of Environment web site (ide.mma.gob.cl). Considering the entire set used in this study (n = 510), the average size of protected areas was 40,218 ha, varying from a minimum of 0.64 ha to a maximum of 3,677,849 ha. All of the seven protected area groups, except PSBC and private protected areas, are listed under an IUCN category [32]. These management categories differ in the level of human activity allowed, from strict protection where no extractive activity is allowed (IUCN Ia- III) to a more permissive approach where human habitation and sustainable extractive use are accepted (IUCN IV-VI) (see Table S1 for details).

**Figure 1:**
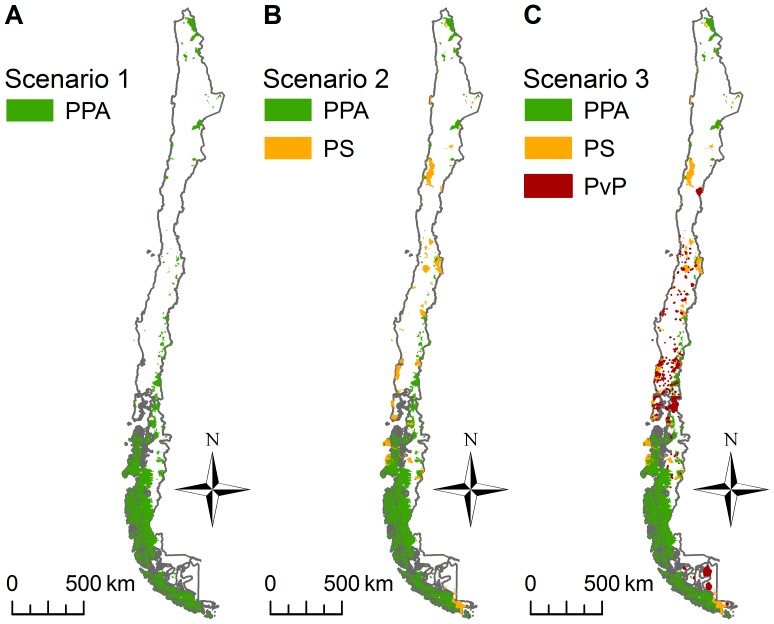
Distribution of three protection scenarios. The scenarios represent alternative conservation approaches. (A) Scenario 1, (B) Scenario 2, (C) Scenario 3. PPA: Public Protected Areas (current PA system in Chile); PS: Priority Sites for Biodiversity; PvP: Private Protected Areas.

Following a similar approach to that of Pliscoff and Fuentes-Castillo [33], we created three protection scenarios in order to evaluate the effectiveness of the existing protection system and the potential contribution of PSBC and private protected areas to the new integrated protected area system. The three scenarios were (Fig. 1):

– Scenario 1 (current protection system, Fig. 1-A): SNASPE+National Sanctuary+Ministry of Heritage lands.
– Scenario 2 (Fig. 1-B): Scenario 1+ Priority sites for biodiversity conservation (PSBC).
– Scenario 3 (Fig. 1-C): Scenario 2+ Private protected areas.

### Distribution of Ecosystem Services and Biodiversity

#### Carbon storage

We calculated carbon storage by combining an estimate of above and below ground vegetation biomass and a soil organic carbon (SOC) dataset (in kg C). Aboveground vegetation data were obtained following the IPCC GPG Tier-1 method for estimating vegetation carbon stocks using the global default values provided for above ground biomass [34]. Below ground vegetative biomass (root) carbon stock was added using the root-to-shoot ratios for each vegetation type (i.e. shrubland, forest, grassland, steppe) obtained from the same IPCC [34] document, and then total living vegetation biomass was converted to carbon stock using the carbon fraction for each vegetation type. All estimates and conversions were specific to each of the nine ecofloristic zones [35] in Chile, and vegetation type obtained from the Chilean land use cover at 1.56 km^2^ resolution (CONAMA). Thus, a total of 246 carbon zones with unique carbon stock values were compiled based on the IPCC Tier-1 methods.

Soil carbon density data were obtained from the most recent soil carbon database, the Harmonized World Soil Database (HWSD) version 1.1 [36], at 1×1 km resolution.

We used these datasets to create a final carbon storage estimation at 1.25×1.25 km resolution.

#### Plant productivity

Plant productivity (PP) patterns were determined using the Normalized Difference Vegetation Index (NDVI), which has been widely used for this purpose [16], [37]–[40]. NDVI is a linear estimator of the fraction of photosynthetically active radiation intercepted by vegetation (fAPAR) [41]–[43], which is the main control of carbon gain (Monteith, 1981) and hence a good estimator of PP. NDVI is derived from the red:near-infrared reflectance ratio [NDVI = (NIR−RED)/(NIR+RED), where NIR and RED are the amount of near-infrared and red light, respectively, reflected by the vegetation and captured by the sensor of the satellite]. The formula is based on the fact that chlorophyll absorbs RED (fAPAR as mentioned above), whereas the mesophyll leaf structure scatters NIR. NDVI values range from −1 to +1, where negative values correspond to an absence of vegetation (e.g. water bodies) and values closer to +1 correspond to abundant and dense vegetation (e.g. evergreen forest).

Monthly NDVI composites were obtained from the 1 km^2^ resolution Global MODIS (TERRA) (Moderate Resolution Imaging Spectroradiometer - LPDAAC, NASA) dataset, available for 2000–2010. For each pixel we calculated the average of the annual NDVI mean for the 10 year period.

#### Agricultural production

Agricultural production was calculated as the sum of gross production (USA dollar) for 2000. In order to generate a fine resolution layer, a spatial disaggregation process was carried out, in which a coarse resolution dataset is ‘disaggregated’ in a finer and related resolution dataset. Specifically, the agricultural production layer was calculated as follows (i) We multiplied the harvested area of 32 major crops (i.e. proportion of a grid cell that has been harvested for a specific type of crop) (Table S2) at 10 km × 10 km resolution [44] by crop land cover at 1 km × 1 km resolution (i.e. spatial distribution of agricultural lands) [45]. Thus, through the disaggregation process, we obtained a second 1 km resolution layer showing the area per pixel (i.e. ha) that was harvested for each major crop; (ii) The resultant layers for each major crop were then multiplied by their respective yields (tonnes/ha) [46], obtaining tonnes of crops produced per pixel; and (iii) Finally, tonnes per pixel of each major crop were then multiplied by prices (USD/tonnes) for 2000 (FAOStat, http://faostat3.fao.org/home/index.html), thus obtaining USD of agricultural production per pixel.

#### Biodiversity

The Chilean biodiversity dataset comprised four taxonomic groups: mammals (n = 113), birds (n = 364), amphibians (n = 58) and vascular plants (n = 1,061). Distribution maps for mammals, birds and amphibians that occur in Chile were obtained from the IUCN Global Mammal Assessment, BirdLife International and the Global Amphibian Assessment, respectively. All these are freely available at the IUCN Red List web site [47], and released as polygon vector files. The dataset for plant distributions was obtained from work carried out by the Ministry of the Environment [48]. Plant distributions were generated using the Maximum Entropy Model (MaxEnt), which was based on a dataset comprising georeferenced records from the largest plant collection in Chile (Museum of Concepción) complemented with records derived from available literature. Only species with more than 10 records entered into the analysis. MaxEnt models were developed using the meteorological database for Chile (1961–1990) developed by the Department of Geophysics of the University of Chile [49]. Plant species distributions were modelled using the variables temperature (max., min. and average), precipitation (max., min. and total), altitude, slope and aspect. The area under the curve (AUC), a criterion used to assess fit in distribution models such as MaxEnt (see [50], was on average 0.978.

Each taxonomic group was analysed separately, using a 1 km × 1 km grid resolution.

### Data Analyses

#### Quantification of ecosystem services and biodiversity within protected areas

A spatial overlap analysis was used to calculate the representation of each ecosystem service and of biodiversity within the Chilean protected area system. The three protection scenario covers were overlapped with each ecosystem service and biodiversity layer, and the spatially coincident coverage extracted. However, as ecosystem service layers and biodiversity were mapped in different units, their representation was calculated in distinct ways as follows:

– The units of carbon storage and agricultural production layers are the total amount of carbon (kg) and USD production, respectively, per pixel. Thus, the representation of these two ecosystem services was calculated as the sum of all those pixels that fell within protected areas.
– The PP captured was estimated from the average of the NDVI values of those pixels that fell within protected areas. As NDVI varies according to vegetation type, we calculated a weighted average in accordance with the proportion of total area of each vegetation type found within the protected area system. Thus, the resulting NDVI average is representative of the extents of different vegetation types within the protected area system. Vegetation types found within protected areas were Forest, Shrubland, Steppe, Wetland, Crop, Peatland and Bare areas (Table S5). For comparison purposes, a weighted NDVI average was also calculated for the entire country (Table S3).
– The representation of biodiversity was calculated as the summed proportion of species’ ranges that fell within the protected area coverage. This was calculated per taxon and for all species together.

#### Assessing effectiveness of protection scenarios and protected area categories

We divided the percentage of each of the measures of ecosystem services and biodiversity contained within each scenario and protected area category by the percentage land area covered by that particular scenario and category [17]. This approach will indicate whether the amount of a given ecosystem service or biodiversity is more or less than would be expected for the protected coverage area. A value greater than one thus indicates that a particular scenario or category contains a disproportionately large amount of a specific ecosystem service or biodiversity group relative to the area that it covers. Our measure of biodiversity within each of the three protection scenario and seven management categories was the summed proportion of the ranges of all species. NDVI is an index and it is thus meaningless to use the same approach, so we calculated a weighted average of NDVI values that fall within each of the seven protected area categories in the same way as indicated above.

## Results

**Figure 2:**
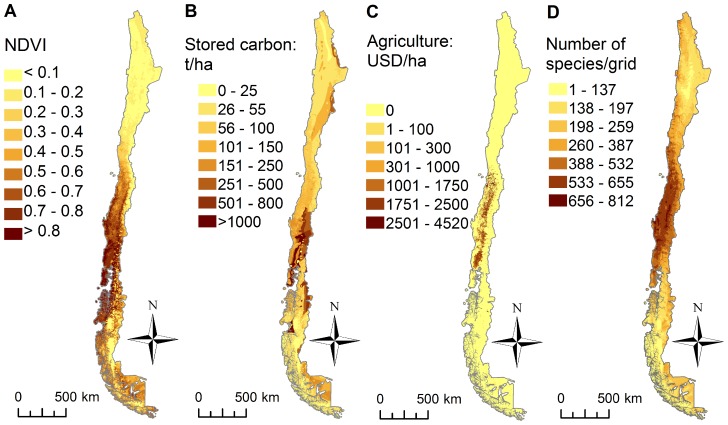
Ecosystem service and biodiversity distribution in Chile. Distribution of (A) net primary production, (B) carbon storage, (C) agricultural production and (D) biodiversity.

The bulk of carbon storage, net primary production and agricultural production were located in the south-central zone of Chile (Fig. 2). Areas with the highest density of stored carbon were located between 36°–41° S, mainly concentrated in the eastern forest (Fig. 2B). Areas with the highest values of NDVI were located between 35°–43° S, particularly in the southern-central coastal range (Fig. 2A). Croplands were grouped in the central valley of Chile between 32°–41° S, and the highest production crops were in the region of Bernardo O’Higgins (32°–34° S) (Fig. 2C). The latitudinal region with the highest species richness was between 31°–40° S (Fig. 2D, Fig.S1).

The proportion of stored carbon varied from a low of 14.9% in Scenario 1, which increased to 19.0% and 19.9% in Scenario 2 and Scenario 3, respectively (Table 1). In the three scenarios carbon was underrepresented as would be expected for their coverage area (i.e. ratio less than 1, Table 1). An NDVI value of 0.38 was represented within the current protected area system (Scenario 1), 0.04 units higher than the whole country average (Table S3). This increased to 0.39 in Scenario 3 when private protected areas were added to the total coverage (Table 1). Only 0.2% of the total agricultural production was captured within the current protected area system (Table 1). However, this representation increased to 2.2% in Scenario 2 and 2.7% with Scenario 3 (Table 1). Again, none of the three representations was as much as would be expected for the area covered by the scenarios (Table 1).

**Table 1:** Provision of ecosystem services and biodiversity under three protection scenarios.

	Scenario 1		Scenario 2		Scenario 3	
	% oftotal	ratio	% oftotal	ratio	% oftotal	ratio
PP^a^		0.38		0.38		0.39
Carbon	14.9	0.73	19.0	0.75	19.9	0.76
Agriculture	0.2	0.01	2.2	0.9	2.7	0.1
Biodiversity	11.8	0.59	17.3	0.68	18.0	0.69
Mammals	13.9	0.69	18.9	0.74	19.7	0.75
Birds	18.2	0.91	23.0	0.91	24.0	0.92
Amphibians	20.7	**1.03**	27.1	**1.07**	27.8	**1.06**
Plants	8.9	0.44	14.7	0.58	15.4	0.59

A ratio of >1 (in bold) indicates that an ecosystem service is over-represented compared with what would be expected for the area; values <1 indicate under-representation. The percentage of the total ecosystem services and biodiversity (summed proportion of ranges) in each of the three scenarios is given. Scenario 1: current protection system; Scenario 2: scenario 1+ suggested priority sites for biodiversity conservation; Scenario 3: scenario 2+ suggested private protected areas.

^a^Weighted average of NDVI pixels within protected areas (see methods).

The current protected areas capture 13.9%, 18.2%, 20.7% and 8.9% of mammal, bird, amphibian and plant ranges respectively. Amphibians was the only group well represented, with a ratio slightly higher than one (1.03). All species’ representation levels increased substantially in Scenario 2 (i.e. when PSBC sites were included), capturing this time 18.9%, 23.0%, 27.1% and 14.7% of mammal, bird, amphibian and plant species’ ranges respectively. In Scenario 2 only amphibian representation ratio was above one (1.07). When Private Protected Areas were included in Scenario 3, representation levels increased by approximately 1% for all species groups, with only that of amphibians’ being well represented (Table 1). When species groups were assessed all together, its level of representation was 11.8%, 17.3% and 18.0% in scenario 1, 2 and 3 respectively. In all scenarios biodiversity was underrepresented (Table 1).

Carbon storage was well represented only by PSBC (4.08 times as much as would be expected for the area). The other protected area categories, except private protected areas, had values slightly below 1 (Table 2). All protected area categories together under-represented carbon stock with a value below 1 (Table 2). Private protected areas had the highest NDVI average value (0.54), followed by National Reserves (0.48), Nature Sanctuaries (0.47), and Ministry of Heritage lands (0.45). PSBC, National Parks and Natural Monuments had NDVI values below 0.4, Natural Monuments having the lowest average (Table 2). All categories had an NDVI value (0.39) slightly higher than the national average (0.38). Agricultural production was also well represented only by PSBC (2.09). This time the rest of the categories, including all categories together, had ratios below 1 (Table 2). Finally, biodiversity, the summed proportion of ranges of all species, was under-represented in all categories together, but was over-represented by Natural Monuments and PSBC categories (Table 2). When the species groups were assessed separately, amphibians were best represented by different protected area categories: Ministry of Heritage lands (2.36), National Parks (1.06), Nature Sanctuaries (2.92), and PSBC (5.46). Amphibians were the only group well represented by all categories together (Table S4). Mammals were well represented by Natural Monuments (1.36), Nature Sanctuaries (1.22), and PSBC (4.05). Birds and plants were over-represented only by PSBC (4.16 and 4.69 respectively), this being the most successful category in the representation of biodiversity (Table S4).

**Table 2:** Provision of ecosystem services and biodiversity under seven protected area categories.

Protected areacategories	Carbonstorage	PP*	Agriculture	Biodiversity
Natural Monument	0.88	0.17	0.06	**1.13**
National Parks	0.75	0.33	0.006	0.55
National Reserve	0.97	0.48	0.02	0.59
Nature Sanctuary	0.75	0.47	0.12	0.88
Ministry of Heritage lands	0.84	0.45	0.0003	0.67
PSBC	**4.08**	0.39	**2.09**	**4.51**
Private PAs	0.37	0.54	0.22	0.21
All PA categories	0.76	0.39	0.10	0.69

A ratio of >1 (in bold) indicates that an ecosystem service is over-represented compared with what would be expected for the area; values <1 indicate under-representation. The percentage of the total amount of biodiversity (summed proportion of ranges) and other ecosystem services in Chile is given for each protected area category. PSBC: Priority sites for biodiversity conservation; PAs: protected areas; ‘All PA categories’ refers to the area covered by all seven categories.

*Weighted average of NDVI values that fall within each of the seven protected area categories (see methods for details).

## Discussion

Previous assessments of the effectiveness of the Chilean protected area system have focused exclusively on biodiversity [7], [27], [33], [51], [52]. Here, we document for the first time the effectiveness of the system in capturing both ecosystem services and biodiversity relative to its area of coverage (Table 1). We found that existing protected areas in Chile do not contain an unusually high proportion of the total national carbon storage (14.9%), agricultural production (0.2%) or species’ ranges (11.8%). Also, PP representation (0.38) was low with regard to the maximum value range (−1 to +1) and with respect to the national forest cover PP (0.63, Table S3). This was, however, slightly higher than the national average (0.34). When the levels of representation were assessed relative to the percentage of land area covered by existing protected areas, we found that amphibians was the only conservation feature overrepresented. The underrepresentation by existing protected areas seems to result from the strong spatial bias of current protected areas toward southern Chile (Fig. 1-A), which raises three key points regarding the resulting representation of ecosystem services and because of their relatively small geographic ranges.

First, as forest coverage, as well as protected areas, is concentrated in southern Chile, we would have expected a higher representation of carbon storage (Table 1). The c. 15% of carbon storage represented reflects, therefore, that southern protected areas are mainly protecting lands devoid of vegetation, such as ice and rock (Table S5). This is also reflected in the level of PP represented within protected areas, which despite being slightly higher than the national average, is closer to zero than to one, indicating a predominance of poorly vegetated lands within the Chilean protected area system.

Second, the underrepresented crop production found within the existing protected areas suggests that these are displacing or avoiding areas of agricultural production, which could reasonably be argued as reflecting their effectiveness. Chilean protected areas conserve a significant proportion of untransformed landscape, facing the challenge of displacing human activities beyond their boundaries. What is not clear however is whether the low agricultural activity within protected areas is due to the management strategy of conserving these lands intact, or because the spatial bias of protected areas towards southern regions renders them unsuitable for agriculture.

Third, while the largest coverage by protected areas is concentrated in the Austral Chilean zone (44°–56° S), our results show that the highest species richness areas are located in the central (28°–36° S) and south-central (36°–43° S) zones of Chile (Fig. S1, see [53]), which is reflected in the underrepresentation of biodiversity within the current protected area system. In fact, central and south-central zones include a hotspot of global biodiversity [54], which is characterized by a large number of endemic plants and vertebrate species [29], [51]. However, amphibians is the only group overrepresented, likely because a relatively high proportion of their distribution ranges covers southern areas.

Adding PSBC and private protected areas to the current protected area system (i.e. Scenarios 2 and 3) enhances the representation of ecosystem services and total biodiversity (Table 1). This increase, however, was not sufficient to attain a representation higher than would be expected for their respective areas of coverage (Table 1). Interestingly, PSBC increase the representation of both carbon storage and crop production, suggesting that current croplands are located in rich organic carbon soil areas, an important proportion of the total calculated carbon storage (see methods). Given that PSBC represent multiple ecosystem services and biodiversity, a multi-goal management strategy will be required in order to optimize the supply of carbon storage and biodiversity as much as agriculture. Thus, conservation planning exercises that include both biodiversity and ecosystem services [55] may be required to improve the Chilean PA network.

When protected areas were evaluated based on their management objective categories, our results showed that no existing protected area category with statutory protection represents the level of ecosystem services and biodiversity one would have expected based on their coverage, except the Natural Monument category that over-represented biodiversity (Table 2). This over-representation is likely related to the small coverage of the Natural Monument category, the smallest of all categories (Tables S6). PSBC was the only category with no statutory protection that over-represented carbon storage (4.08 times as much as would be expected for their area), agricultural production (2.09 times) and biodiversity (4.51 times) (Table 2). Despite the under-representation of existing protected area categories together, our results show that National Parks, the strictest protection category (IUCN, Ia), represent a carbon ratio close to one (0.75), and a very low representation value for agriculture (0.006), which is also reflected in the proportion of land use cover within this category (Table S6). This is consistent with the strict and single land use management aim of this category, which is apparently mainly promoting carbon storage. By contrast, Nature Sanctuary sites, the more permissive category (IUCN, VI), represent exactly the same ratio of carbon storage as National Parks, but also 20 times more crop production, indicating the multi-use landscape nature of this category (Table S6). Only Natural Monument and PSBC categories over-represent biodiversity, indicating that these protected area categories are well placed with regard to species’ range distributions (Table 2), however around 20% of amphibian are gap species, not yet represented in protected areas [29]. When species groups were assessed separately, amphibians was the only one overrepresented by all protected area categories (1.06), although the bird representation ratio was very close to one (0.92) (Table S4).

The existing Chilean protected area network does not perform well in representing all biodiversity groups together, but achieves a good representation of amphibians. Also, its provision of ecosystem services is poor. It is highly likely that this gap would need to be addressed principally by the expansion of the coverage of the protected area system. In this regard we suggest two measures. First, a re-evaluation of the already suggested new sites for the integrated protected area system, as these do not significantly increase ecosystem service representation. Second, a systematic assessment plan of current conservation management objectives and strategies, in order to enhance ecosystem service supply by existing protected areas.

## Supporting Information

Figure S1 **Distribution maps of species richness for four taxonomic groups at 1** **km^2^ grid resolution.** a) Amphibians, b) Mammals, c) Birds and d) Plants.
(TIF)

Table S1 **Protected area categories used in this study, and their associated management strategies defined under the International Union for Conservation of Nature (IUCN) regulatory framework.**
(DOC)

Table S2 **Summary of values used in calculating agricultural production (FAO, 2000).**
(DOC)

Table S3 **Average of NDVI values and coverage characteristics of different vegetation types in Chile.**
(DOC)

Table S4 **Biodiversity representation by species group in the five management categories and the suggested sites for the new integrated protection system (PSBC and Private protected areas).** A ratio of >1 indicates that a particular group is over-represented relative to what would be expected for its area; values <1 indicate under-representation. ‘All management strategies’ refers to the area covered by all the seven categories. PA: Protected Area; PSBC: Priority sites for biodiversity conservation.
(DOC)

Table S5 **Land use cover within the current Chilean protected areas system (Scenario 1).**
(DOC)

Table S6 **Land cover within each of the five management categories and the suggested sites for the new integrated protection system (PSBC and Private protected areas).** PA: Protected Area; PSBC: Priority sites for biodiversity conservation.
(DOC)
