# Supplementary figures and images for: Representation of Ecosystem Services by Terrestrial Protected Areas: Chile as a Case Study
Source: PLoS One. 2013 Dec 20;8(12):e82643. doi: 10.1371/journal.pone.0082643 (PMC3869732; doi:10.1371/journal.pone.0082643)

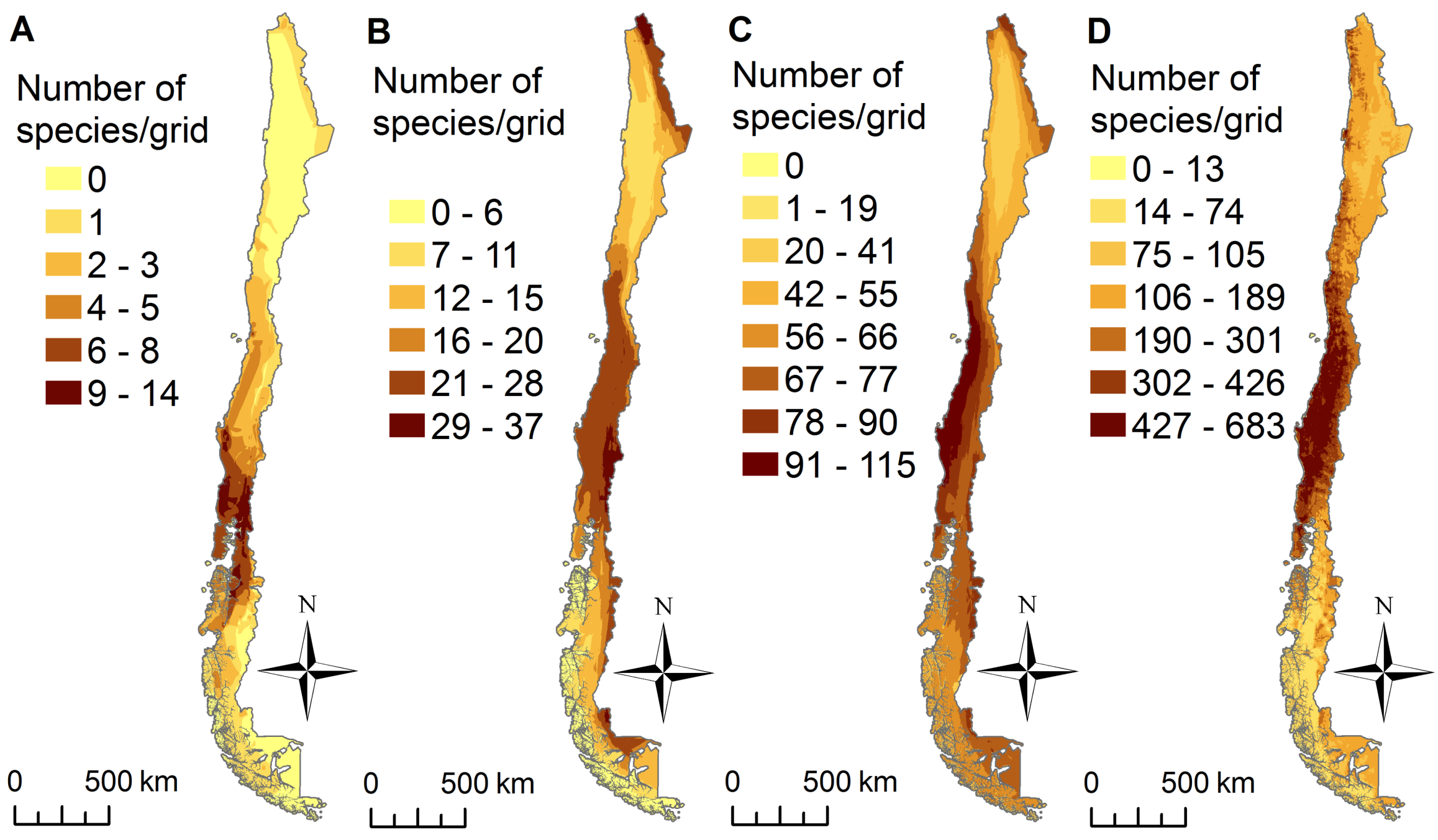

Supplement: Figure S1 — Distribution maps of species richness for four taxonomic groups at 1 km2 grid resolution. a) Amphibians, b) Mammals, c) Birds and d) Plants. (TIF) [file pone.0082643.s001.tif]
