# Supplementary material for: Representation of Ecosystem Services by Terrestrial Protected Areas: Chile as a Case Study
Source: PLoS One. 2013 Dec 20;8(12):e82643. doi: 10.1371/journal.pone.0082643 (PMC3869732; doi:10.1371/journal.pone.0082643)
Supplement: Table S1 — Protected area categories used in this study, and their associated management strategies defined under the International Union for Conservation of Nature (IUCN) regulatory framework. (DOC) [file pone.0082643.s002.doc]

**Table S1** Protected area categories used in this study, and their associated management strategies defined under the International Union for Conservation of Nature (IUCN) regulatory framework.

| Protected area category | Institution administrator | Management aims | IUCN  category |
| --- | --- | --- | --- |
| National Park (SNASPE) | CONAF | Protection and conservation of natural scenic beauty, flora and fauna. Only scientific and educational activities are allowed. | II |
| National Reserve  (SNASPE) | CONAF | Conservation through managed intervention of natural resources | IV |
| Natural Monument  (SNASPE) | CONAF | Protection of a specific natural feature with aesthetic, historical or scientific value. | III |
| Nature sanctuary | National Monument Board | Conservation for scientific or government purpose. | V |
| Lands of national heritage | Ministry of National Heritage | Conservation of natural ecosystem and national patrimony. Sustainable management of natural resources are allowed. | IV |
| Priority Sites for Biodiversity Conservation | CONAMA | NA | No category |
| Private Protected Areas | Private | NA | No category |
