# Supplementary material for: Representation of Ecosystem Services by Terrestrial Protected Areas: Chile as a Case Study
Source: PLoS One. 2013 Dec 20;8(12):e82643. doi: 10.1371/journal.pone.0082643 (PMC3869732; doi:10.1371/journal.pone.0082643)
Supplement: Table S2 — Summary of values used in calculating agricultural production (FAO, 2000). (DOC) [file pone.0082643.s003.doc]

**Table S2** Summary of values used in calculating agricultural production (FAO, 2000)

| Crop | USD/tonne |
| --- | --- |
| Alfalfa | 80 |
| Apples | 196.4 |
| Appricot | 262.8 |
| Artichok | 374.9 |
| Asparragus | 658.4 |
| Avocado | 1132.7 |
| Barley | 165.3 |
| Carrot | 145.5 |
| Cherry | 810.6 |
| Chickpea | 639.4 |
| Grape | 230.2 |
| Green bean | 342.8 |
| Kiwi | 162.7 |
| Lemon | 238.6 |
| Lentils | 443.5 |
| Lettuce | 329.8 |
| Maize | 127.6 |
| Oats | 120.5 |
| Onion | 276.6 |
| Orange | 164.4 |
| Pea | 346.1 |
| Peach | 293.6 |
| Pear | 224.4 |
| Plum | 163.2 |
| Potato | 155.7 |
| Pumpkin | 165.8 |
| Sugar beet | 55.4 |
| Sunflower | 237.2 |
| Tobacco | 1679.3 |
| Tomato | 302.9 |
| Watermelon | 181.8 |
| Wheat | 95.2 |
