# Supplementary material for: Representation of Ecosystem Services by Terrestrial Protected Areas: Chile as a Case Study
Source: PLoS One. 2013 Dec 20;8(12):e82643. doi: 10.1371/journal.pone.0082643 (PMC3869732; doi:10.1371/journal.pone.0082643)
Supplement: Table S3 — Average of NDVI values and coverage characteristics of different vegetation types in Chile. (DOC) [file pone.0082643.s004.doc]

**Table S3** Average of NDVI values and coverage characteristics of different vegetation types in Chile.

| Vegetation type | NDVI average | Area coverage (km^2^) | Proportion of area | Weighted average  (NDVI average x proportion of area) |
| --- | --- | --- | --- | --- |
| Forest | 0.627 | 191,704 | 0.253 | 0.158 |
| Crops | 0.564 | 8,803 | 0.012 | 0.006 |
| Peatland | 0.487 | 19,708 | 0.026 | 0.012 |
| Steppe | 0.346 | 43,945 | 0.058 | 0.02 |
| Shrubland | 0.341 | 152,203 | 0.201 | 0.068 |
| Wetland | 0.177 | 1,129 | 0.001 | 0.0002 |
| Bare areas^(1)^ | 0.174 | 338,799 | 0.447 | 0.078 |
|  |  |  | **Total NDVI average** | **0.34** |

^(1)^ Bare areas category includes iceland, rock and sand.
