# Supplementary material for: Representation of Ecosystem Services by Terrestrial Protected Areas: Chile as a Case Study
Source: PLoS One. 2013 Dec 20;8(12):e82643. doi: 10.1371/journal.pone.0082643 (PMC3869732; doi:10.1371/journal.pone.0082643)
Supplement: Table S4 — Biodiversity representation by species group in the five management categories and the suggested sites for the new integrated protection system (PSBC and Private protected areas). A ratio of >1 indicates that a particular group is over-represented relative to what would be expected for its area; values <1 indicate under-representation. ‘All management strategies’ refers to the area covered by all the seven categories. PA: Protected Area; PSBC: Priority sites for biodiversity conservation. (DOC) [file pone.0082643.s005.doc]

**Table S4** Biodiversity representation by species group in the five management categories and the suggested sites for the new integrated protection system (PSBC and Private protected areas). A ratio of > 1 indicates that a particular group is over-represented relative to what would be expected for its area; values < 1 indicate under-representation. 'All management strategies' refers to the area covered by all the seven categories. PA: Protected Area; PSBC: Priority sites for biodiversity conservation.

| PA  Category | Amphibians | Mammals | Birds | Plants |
| --- | --- | --- | --- | --- |
| Ministry of Heritage lands | **2.36** | 0.90 | 1.00 | 0.50 |
| National Parks | **1.06** | 0.72 | 0.92 | 0.39 |
| National Reserve | 0.76 | 0.56 | 0.83 | 0.51 |
| Natural Monument | 0.72 | **1.36** | 0.98 | 0.84 |
| Nature Sanctuary | **2.92** | **1.22** | 1.09 | 0.75 |
| PSBC | **5.46** | **4.05** | **4.16** | **4.69** |
| Private PA | 0.43 | 0.25 | 0.25 | 0.18 |
| All management strategies | **1.06** | 0.75 | 0.92 | 0.59 |
