# Supplementary material for: Representation of Ecosystem Services by Terrestrial Protected Areas: Chile as a Case Study
Source: PLoS One. 2013 Dec 20;8(12):e82643. doi: 10.1371/journal.pone.0082643 (PMC3869732; doi:10.1371/journal.pone.0082643)
Supplement: Table S5 — Land use cover within the current Chilean protected areas system (Scenario 1). (DOC) [file pone.0082643.s006.doc]

**Table S5** Land use cover within the current Chilean protected areas system (Scenario 1).

| Vegetation type | Percentage of total area |
| --- | --- |
| Forest | 34.15 |
| Crops | 0.0007 |
| Peatland | 9.48 |
| Steppe | 3.1 |
| Shrubland | 2.72 |
| Wetland | 0.38 |
| Bare areas^(1)^ | 50.14 |

^(1)^ Bare areas category includes iceland, rock and sand.
