# Supplementary material for: Representation of Ecosystem Services by Terrestrial Protected Areas: Chile as a Case Study
Source: PLoS One. 2013 Dec 20;8(12):e82643. doi: 10.1371/journal.pone.0082643 (PMC3869732; doi:10.1371/journal.pone.0082643)
Supplement: Table S6 — Land cover within each of the five management categories and the suggested sites for the new integrated protection system (PSBC and Private protected areas). PA: Protected Area; PSBC: Priority sites for biodiversity conservation. (DOC) [file pone.0082643.s007.doc]

**Table S6** Land cover within each of the five management categories and the suggested sites for the new integrated protection system (PSBC and Private protected areas). PA: Protected Area; PSBC: Priority sites for biodiversity conservation.

|  | Percentage of area | | | | | | |  |
| --- | --- | --- | --- | --- | --- | --- | --- | --- |
| PA  Category | Forest | Crops | Peatland | Steppe | Shrubland | Wetland | Bare areas^(1)^ | **Coverage area (km^2^)** |
| Ministry of Heritage lands | 43.58 | 0 | 11.37 | 2.13 | 9.48 | 0 | 33.31 | 1,796 |
| National Parks | 30.17 | 0 | 7.62 | 2.91 | 2.15 | 0.33 | 56.8 | 93,104 |
| National Reserve | 39.39 | 0 | 13.76 | 3.58 | 2.67 | 0.48 | 40.09 | 52,824 |
| Natural Monument | 7.4 | 0 | 0 | 9.21 | 10.93 | 7 | 65.44 | 381 |
| Nature Sanctuary | 53.75 | 0.002 | 0 | 2.17 | 11.49 | 0 | 32.60 | 4,594 |
| PSBC | 30.33 | 0.28 | 1.88 | 3.22 | 35.21 | 0.06 | 29.02 | 42,591 |
| Private PA | 48.18 | 0.45 | 0.23 | 2.1 | 29.63 | 0.06 | 19.33 | 9,867 |

^(1)^ Bare areas category includes iceland, rock and sand.
